# Extended risk-analysis model for activities of the project

**DOI:** 10.1186/2193-1801-2-227

**Published:** 2013-05-17

**Authors:** Janez Kušar, Lidija Rihar, Urban Žargi, Marko Starbek

**Affiliations:** Faculty of Mechanical Engineering, Aškerčeva 6, SI-1000 Ljubljana, Slovenia; TCG Unitech LTH-ol, Vincarje 2, Škofja Loka, Slovenia

**Keywords:** Project management of orders, Project risks, Activity risks, Status indicators

## Abstract

Project management of product/service orders has become a mode of operation in many companies. Although these are mostly cyclically recurring projects, risk management is very important for them. An extended risk-analysis model for new product/service projects is presented in this paper. Emphasis is on a solution developed in the Faculty of Mechanical Engineering in Ljubljana, Slovenia. The usual project activities risk analysis is based on evaluation of the probability that risk events occur and on evaluation of their consequences. A third parameter has been added in our model: an estimate of the incidence of risk events. On the basis of the calculated activity risk level, a project team prepares preventive and corrective measures that should be taken according to the status indicators. An important advantage of the proposed solution is that the project manager and his team members are timely warned of risk events and they can thus activate the envisaged preventive and corrective measures as necessary.

## Introduction

**Figure 1:**
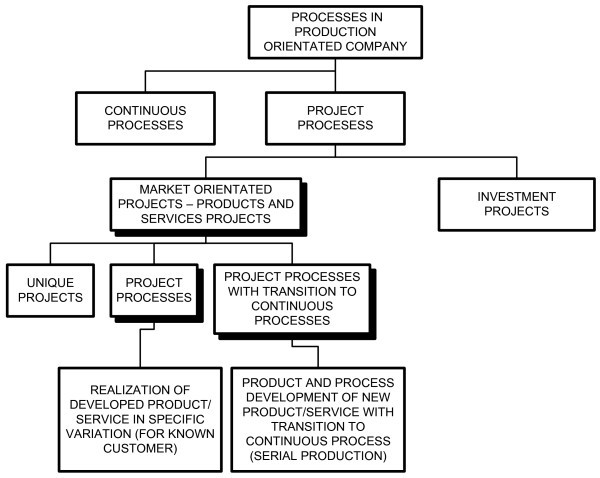
**Processes in a manufacturing company.**

Mass production was the prevailing production concept until the end of the 20th century, while today companies favour a transition to a project type of production (Kendall & Rollins 2003). This is the case not only in companies that manufacture special equipment for new investments – this transition can also be seen in companies that have traditionally been using continuous mass production, e.g. in the automotive industry (Fleischer & Liker 1997). Companies today, therefore, have to deal simultaneously with continuous and project processes (Figure 1).

Continuous processes are carried out for an “indefinite period of time” for providing new quantities of previously developed products (according to market demand).

Project processes are carried out once or in standard repetitions; they are aimed at achieving a precisely defined objective, for a known customer, and their duration is limited.

Project processes can be either internal or market-oriented. Internal projects are aimed at development of the company, e.g. research of new markets, infrastructure improvements or business process re-engineering.

Market-oriented projects can be one-time projects (e.g., building a new manufacturing facility), cyclically recurring projects (e.g., manufacturing a turbine) or new-product-development projects with subsequent transition to continuous production (e.g., development of a car pedal component).

This article deals only with market-oriented projects arising from known-customer orders. The orders express wishes for supply or delivery of products or services.

In spite of the fact that project processes are recurring, project risk management is very important, because these projects are very precisely defined in terms of deadlines, costs and quality. Any discrepancy from the project plan can lead to business and competitive loss. At the start of the project, the customer and the company jointly take a risk on successful project implementation and on good market penetration of the product.

Companies often fear that projects would be paralysed by making risk analysis, or even that identifying risks would frighten them so much that they would not carry out the project. However, risk management brings several benefits to a company:

- Organisational benefits: increased project implementation efficiency (fewer errors, corrections and delays).
- Market benefits related to efficiency of projects: the more accurately the required time and cost of the project are calculated, and more effectively the risk is managed, the higher is the profit made by the project and the higher is customer satisfaction.
- Strategic benefits of project risk management are logical if market benefits of several successful projects are summed up, and then a consideration is made of what this means for a company in the long term.

Well-planned project risk management ensures higher customer satisfaction and thus a higher reputation for the company. Forward project management with established mechanism of risk management allows the company to function much more effectively and successfully in times of continuous changes.

Presented below are the results of a project activities risk management model for developing new products or services. The model is based on quantitative risk analysis and on experience of implementing project management in Slovenian companies.

## Project activities risk management model

Activity or project risks (PMBOK Guide 2004; Paul 2002) are possible events or circumstances that can threaten the planned project course. Risk analysis is the most important tool used by project managers in project risk management.

Several models and methods are available for risk analysis of project activities (Cappels 2004; Goodpasture 2004; Smith & Merritt 2002; Vargas 2008; Risk management guide for DOD acquisition 2006; Dobie 2007).

Cappels (Cappels 2004) said that effective risk management requires four steps for assessing and managing risk: identification of potential risks, quantification of risk, developing a response to risk and risk response control. Goodpasture (Goodpasture 2004) using Kano model and project balance sheet for managing of risk. Smith and Merritt (Smith & Merritt 2002) propose proactive risk management in five steps: identify risks, analyze risk, prioritize and map risks, resolve risk and monitor risk. Vargas (Vargas 2008) discuses and analyze the model and best practices used in the PMI standards, published in the PMBOK guide (PMBOK Guide 2004). The risk management processes model (Risk management guide for DOD acquisition 2006) includes the following key activities of risk: identification, analysis, mitigation planning, mitigation plan implementation and tracking. The risk reporting matrix is used to determine the level of risk. Dobie (Dobie 2007) proposes the risk management process in nine steps.

**Figure 2:**
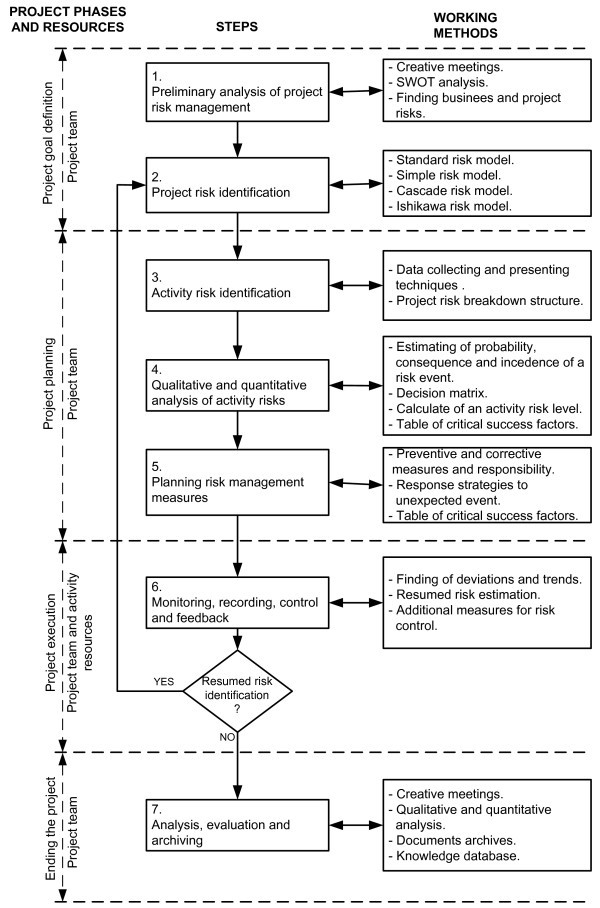
**Project activities risk management model.**

Analysis of available models and methods of project risk management, supported by experience of project implementation in an industrial environment (Kušar et al. 2008), led the researchers of the Laboratory for Manufacturing Systems at the Faculty of Mechanical Engineering in Ljubljana to create a project activities risk management model, shown in Figure 2.

The starting point was a review of the reference model analysis proposed by (Dobie 2007).

Comparison of the two models showed the following differences:

- In reference’s model, risk analysis of project activities is carried out in nine steps:
- step 1: establishing the context,
- step 2: preliminary risk analysis,
- step 3: detailed risk identification,
- step 4: detailed risk analysis,
- step 5: detailed risk evaluation,
- step 6: risk treatment (planning),
- step 7: prepare risk management plan,
- step 8: risk monitoring and control,
- step 9: review.
- In the proposed model, risk analysis of project activities is carried out in seven steps, on the basis of guidelines in (PMBOK Guide 2004):
- step 1: preliminary analysis of project risk management,
- step 2: project risk identification,
- step 3: activity risk identification,
- step 4: qualitative and quantitative analysis of activity risks,
- step 5: planning risk management measures,
- step 6: monitoring, recording, control and feedback,
- step 7: analysis, evaluation and archiving.
- The differences between these two models are presented in Table 1.
- Additionally, the proposed model incorporates the most frequently used work methods that a project team can use to carry out a particular risk analysis step.

**Table 1:** **Overview of differences between the treated risk analysis models**

Steps of the reference’s model	Steps of the proposed model	Differences
1. Establishing the context	1. Preliminary analysis of project risk management	- Steps 1 and 2 of reference’s model are combined in one step in the proposed model
		- Preliminary analysis of the proposed model refers to the whole project
2. Preliminary risk analysis		
3. Detailed risk identification	2. Project risk identification	- Step 3 of reference’s model is logically divided into risk analysis of the whole project and then to detailed analysis of risk activities
	3. Activity risk identification	
4. Detailed risk analysis	4. Qualitative and quantitative analysis of activity risks	- Estimate of risk parameters and evaluation of risk are logically connected
5. Detailed risk evaluation		
6. Risk treatment (planning)	5. Planning risk management measures	- Planning measures is carried out on the basis of risk evaluation, so two steps are not necessary
7. Prepare risk management plan		
8. Risk monitoring and control	6. Monitoring, recording, control and feedback	
9. Review	7. Analysis, evaluation and archiving	- In the proposed model, emphasis is placed on evaluation and formation of knowledge on the basis of experience obtained in the completed project

### Preliminary analysis of project activities risks

The project team holds a creativity workshop in order to identify possible project activity risks in view of strategic, organisational and project goals, and to analyse important project participants and their influence on the risks.

There are project and business risks. Business risks mainly influence the decision on whether it is possible or sensible to carry out the project, while project risks influence decisions on how to carry out a project so that its execution is most effective bearing in mind the objectives and given circumstances.

The project team uses SWOT analysis to carry out this step. SWOT analysis defines strengths, weaknesses, opportunities and threats related to project execution and its risks.

On the basis of the SWOT-analysis results, the project team and the customer can decide either that the risk-level is acceptable (so the project will be carried out), or the risk-level is too high (and the project will not be carried out).

### Project risk identification

The project team can select one of the following models for the identification of project risks (Smith & Merritt 2002):

- Standard model, in which risk is defined with two parameters: risk event and its influence on the course of the project.
- Simple model, in which risk is defined with one parameter that refers to the risk event and its influence.
- Cascade model, in which risk is defined with risk event consequences and influences on the course of the project.
- Ishikawa model, in which sources of project risks and their corresponding risk events are defined. On the basis of this model, the project team identifies the risk sources and events that have the most influence on project implementation.

**Figure 3:**
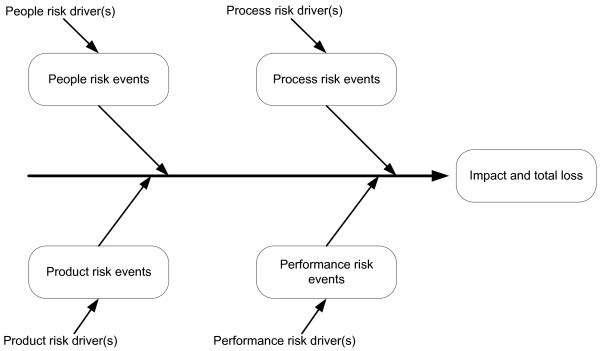
**The Ishikawa model for identification of project risks.**

Analysis of practical use of the listed models has shown that the Ishikawa model (Figure 3) is the most suitable for identification of product/service project risks. The model has the following advantages:

- Companies already know the Ishikawa model as an effective tool for total quality management (TQM).
- The model gives a clear presentation of why project risks occur.
- Separated risk events allow preventive measures.
- The model supports the cause-and-effect concept.

### Identification of project activities risks

For quantitative analysis of project activities risks, the project team can use data collection and presentation techniques, e.g., risk event – incidence, whereby the findings of previously completed similar projects are used, or a project activity risk breakdown structure (RBS) method (PMBOK Guide 2004), (Vargas 2008). The methods listed in section 2.2 can also be used.

**Figure 4:**
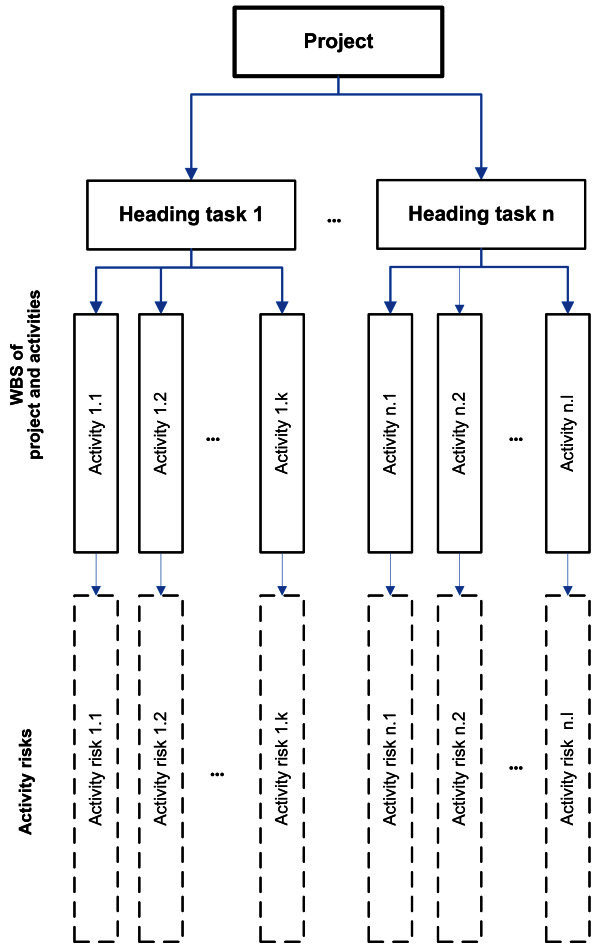
**Breakdown structure of project activity risks.**

The project risk breakdown structure method is the most suitable for practical use. In it, the standard WBS project structure (PMBOK Guide 2004), (Vargas 2008) is extended by risks identified for a particular activity. If it is not possible to identify a risk related to a particular activity, the risk is omitted. A method of breaking down the structure of project activity risks is shown in Figure 4.

### Qualitative and quantitative analysis of project activity risks

Qualitative and quantitative analysis of project activity risks is carried out by evaluating (PMBOK Guide 2004), (Risk management guide for DOD acquisition 2006):

- probability that a problem or risk event will occur
- consequences of a problem or risk event
- definition of risk level.

An interval scale with rates from 1 to 5 can be used to estimate the risk event incidence probability (Risk management guide for DOD acquisition 2006). Another possibility is to use a scale with estimated probability values (PMBOK Guide 2004). A 1-to-5 scale is usually used in practice because of its simplicity.

Values in Table 2 are used for estimating the incidence of a problem or risk event.

**Table 2:** **Probability that a risk event will occur**

Estimate	Event probability - EP
1	very low (~ 10%)
2	low (~ 30%)
3	medium (~50%)
4	high (~ 70%)
5	very high (~ 90%)

Values in Table 3 are used for estimating the consequences of a problem or risk event.

**Table 3:** **Estimate of consequences of a risk event**

Estimate	Estimate of consequences of an event - EC
1	very low
2	low
3	medium
4	high
5	very high

On the basis of the estimated probability that a risk event will occur and on the basis of the estimate of its consequences, project activity risk level is calculated. In two-dimensional analysis, activity risk level is calculated as:1

Where:

*RL*_*2*_ – activity risk level in two-dimensional analysis of project activity risk

*EP* – probability that a risk event will occur

*EC* – estimate of risk event consequences

Data on quantitative and qualitative risk analysis of a particular project activity are entered into a table of critical success factors (Table 4).

**Table 4:** **Table of critical success factors—two-dimensional analysis**

No.	WBS code/Activity/Problem	Event probability EP	Consequence estimate EC	Risk level RL2
1.	Activity 1/Problem A	3	2	6
2.	Activity 2/Problem B	2	4	8
j	Activity j/Problem N	4	5	20
n	Activity n/Problem X	4	5	20

The article deals with risks in cyclically recurring projects, so experience derived from similar past projects can be used for estimating the incidence of risk events.

An example: a company plans the activity of the customer’s confirmation of documentation or product samples. Some time is planned to accomplish this activity. However, the customer often (but not always) exceeds the planned time. In this case there is a recurring risk event.

The researchers from the Laboratory for Manufacturing Systems propose that the values in Table 5 be used for estimating the incidence of problems or risk events.

**Table 5:** **Estimate of risk event incidence**

Estimate	Estimate of event incidence - EI
1	never
2	very rarely
3	rarely
4	often
5	very often

In three-dimensional analysis, project activity risk level is calculated as:2

Where:

*RL*_*3*_ – activity risk level in three-dimensional analysis of project activity risk

*EP* – probability that a risk event will occur

*EC* – estimate of consequences of a risk event

*EI* – estimate of recurring risk event incidence

Table 6 shows critical success factors for three-dimensional risk analysis.

**Table 6:** **Table of critical success factors—three-dimensional analysis**

Risk analysis					
No.	WBS code/Activity/Problem	Event probability EP	Consequence estimate EC	Incidence estimate EI	Risk level RL3
1.	Activity 1/Problem A	3	2	4	24
2.	Activity 2/Problem B	2	4	4	32
j.	Activity j/Problem N	4	5	5	100
n	Activity n/Problem X	4	5	3	60

### Planning measures and risk management

After the risk analysis is completed, activity risk is defined as low, medium or high (on the basis of a decision matrix (PMBOK Guide 2004), (Risk management guide for DOD acquisition 2006)), depending on the estimate of event incidence probability and its consequences.

On the basis of pre-set risk probability limiting values, project activity risk is defined in the proposed three-dimensional risk analysis:

- If *RL* ≤ 24 (risk probability is up to 20%), the risk is low.
- If 25 ≤ RL ≤ 60 (risk probability is between 20 and 50%), the risk is medium.
- If RL ≥ 61 (risk probability is more than 50%), the risk is high.

If the risk is low, the project team does not specify any measures in advance.

If the risk is medium, the project team prepares preventive measures, focused on the elimination of sources of risk events occurring. If the risk event occurs nevertheless, the project team has to prepare corrective measures immediately.

If the risk is high, the project team prepares both preventive measures to prevent the risk event from occurring (elimination of risk, reduction of possibility of risk realisation, transfer of risk) and corrective measures (active management of risks), which may start processes for alleviation of risk-event consequences.

It is proposed that the measures (together with bearers of responsibilities) are entered into Table 7, which is essentially an extension of Table 6.

**Table 7:** **Supplemented table of critical success factors**

Risk analysis						Risk management		
No.	WBS code/Activity/Problem	Event probability EP	Consequence estimate EC	Incidence estimate EI	Risk level RL_3_	Measures	Responsibility	Indicator
						P – preventive		
						K - corrective		
1.	Activity 1/Problem A	3	2	4	24			
2.	Activity 2/Problem B	2	4	4	32	P – Prevent. measure 1	Project manager	
j.	Activity j/Problem N	4	5	5	100	P – Prevent. measure 2	Manager of development	X days delay
						P – Correct. measure 1		
n	Activity n/Problem x	4	4	3	48	P – Measure 3	Project manager	

### Monitoring, recording, control and feedback

The project manager, project team, customer and operators of activities are responsible for project-risk monitoring and for the implementation of measures.

Each risk has its “owner” and his task is to identify the symptom of the occurring risk as soon as possible and to launch the planned measures on time. The sooner the risk is discovered, the smaller are its consequences.

At regular control meetings, the project manager checks the risk status and updates the risk list if necessary. The team must be aware that the risk level changes over time—in some phases one risk is more probable and in other phases other risks are more probable. For better control, it is therefore important that the risks are sorted by size and by their current relevance.

Researchers from the Laboratory for Manufacturing Systems propose several measures for reduction of project risk level:

- active risk management
- removal of risks
- decrease of the probability of a risk occurring
- alleviation of consequences by transferring risk to another organisation
- passive acceptance of risks by providing time and financial reserves.

Active risk management means that an action plan is prepared for if a risk event occurs, and usually time and financial reserves are also foreseen for solving the consequences of realised risks.

Risk can be totally avoided by eliminating or bypassing the cause of a risk occurring. This is possible by changing the project plan, whereby the whole project is changed or just one phase, duration of an activity, the way an activity is carried out, a supplier or operator. The new plan that tries to avoid risk can be defined as an alternative method for achieving key objectives and may cause higher project costs.

Another way of eliminating risk is elimination of some customer requirements that are difficult to achieve and thus represent risks (time, costs, quality). This method of risk elimination requires negotiations with the customer. In the decision-making process, it is necessary to compare risk with yield if customer requirements are fulfilled.

By placing a risk on the risk list, the possibility of the risk event occurring is automatically reduced because of subsequent systematic control. Carefully planned reduction of risk probability can be achieved by additional activities and costs; other possible actions are: using better and more expensive equipment, using better and more expensive manufacturing technology, aid from external experts or simulations made in advance.

When dealing with the reduction of risk consequences, the best solution is to transfer the risk to another organisation. Within the project partners the risk can be transferred to the customer, outsourcer or supplier. Risk transfer details (delays and additional costs) are defined in a contract. Risk bearers want to avoid additional costs and the probability of a risk occurring is thus reduced. Insurance is another way of mitigating consequences. Insurance is the most suitable when the risk is high, its probability is low, but its consequences could be catastrophic.

The more activities there are on the critical path, the more risky is the project, because delays in critical activities directly cause a delay to the whole project. In non-critical activities, time reserves may considerably reduce the risk due to delay of activities.

In practice, MS Project is often used as a tool for project management IT support, so the employees of the Laboratory for Manufacturing Systems of the Faculty of Mechanical Engineering in Ljubljana, together with our partners in companies, decided to build the presented extended project-activity-risk-management methodology into templates. Although it is possible to use a risk-analysis tool in the server version of MS Project, we believe that, from the user’s perspective, the proposed solution is simpler but very effective. This is confirmed by the use of the extended risk analysis in several industrial projects.

### Analysis, evaluation and archiving

After a project has been completed, the project team (in addition to other analyses) evaluates the risk management in order to discover which expected risk events actually occurred, what were their consequences, and how efficient were the preventive and corrective measures.

All risk-management-related documents are archived; the risk knowledge base is also updated.

## Case study of a project risk analysis

**Figure 5:**
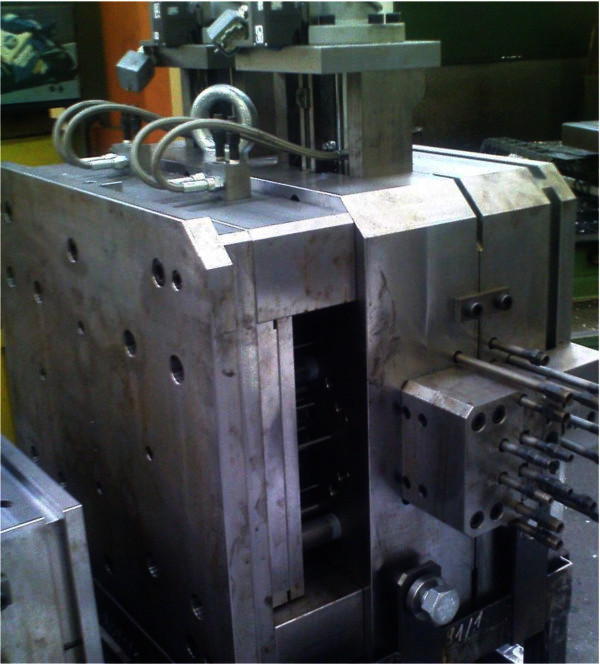
**Die-casting tool—project TL783701.**

A test of the proposed model for project activities risk management was carried out in TCG Unitech LTH-ol company in a project of development and manufacturing a die-casting tool (project TL783701), shown in Figure 5.

The customer sent design documentation, so the order consisted only of process planning and tool manufacturing. The order was completed by confirmation of the product samples. In addition to the development of the die-casting tool, a trimming tool and clamping devices for machining of parts were manufactured within the project.

This was the first time that the company had dealt with project risk analysis, so the company management organised a creativity workshop, the objective of which was to discover all possible risks that could arise in the course of projects in their company, using the Ishikawa model.

**Figure 6:**
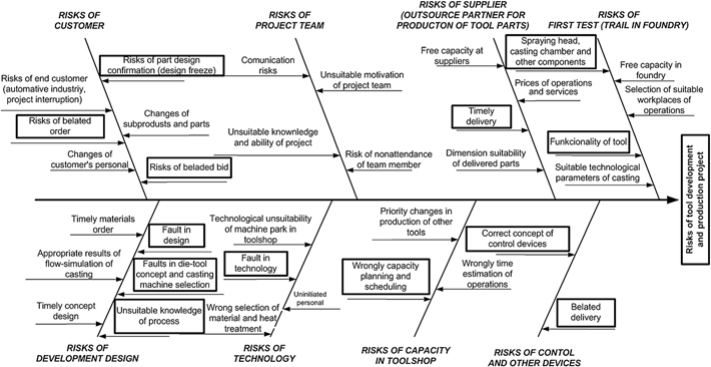
**Cause–effect diagram of risks of project TL783701.**

The result of the creativity workshop was an Ishikawa cause-effect diagram (Figure 6). In it, rectangles denote the causes that may contribute most to the activity risks in this project.

On the basis of the cause-effect diagram the project team reviewed project TL783701 WBS and identified possible risk events for each activity.

**Figure 7:**
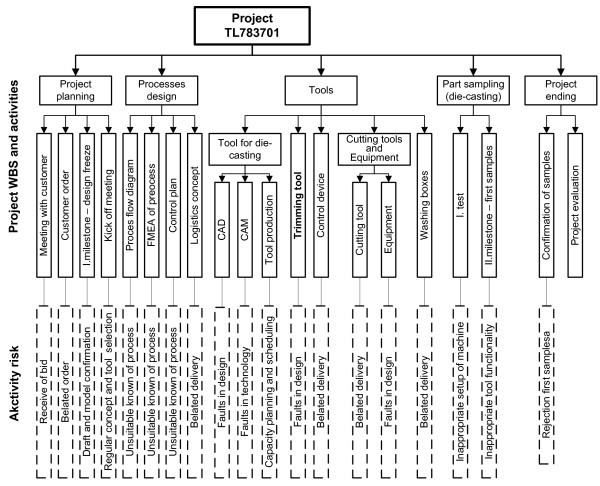
**Risk breakdown structure of project TL783701.**

The project team continuously updated project WBS with the possible activity risks that were found, and transformed WBS to a risk breakdown structure-RBS, as shown in Figure 7.

The project team made a critical success factor table for qualitative and quantitative analysis of project TL783701 activity risks (Table 8). The team members decided to make a three-dimensional risk analysis. They specified for each activity and for its corresponding risk: probability of arising, estimate of consequences, estimate of risk-event incidence, and they calculated the activity risk level. For activities with medium or high risk, they defined preventive and corrective measures and status indicators.

**Table 8:** **Table of critical success factors of TL783701 project (partial)**

Risk analysis						Risk management		
No.	WBS code/Activity/***Problem***	Event probability VD	Consequence estimate OP	Incidence estimate PP	Risk level ST_T_	Measures	Responsibility	Indicator
						P – preventive		
						K - corrective		
1.	**Draft phase/**Meeting with customer/*Accepting bid on time*	3	3	2	18			
2.	**Draft phase/**Client’s order	3	5	1	15			
	*Timely order*							
3	**Design phase**/Milestone I – Design freeze/*Confirmation*	4	5	4	80	**P:** Making a milestone plan	Project manager	Rejection of part drawing
						**K:** Change of schedule	Project manager	
25	/Confirmation of samples/	0	0	0	0			

The critical success factor table allowed them to identify several possible risks for each activity. They used the maximum value as the activity risk level.

MS Project software is used for project planning and control in this company, so the employees of the Laboratory for Manufacturing Systems made a standard MS Project template for the project activity risk management.

**Figure 8:**
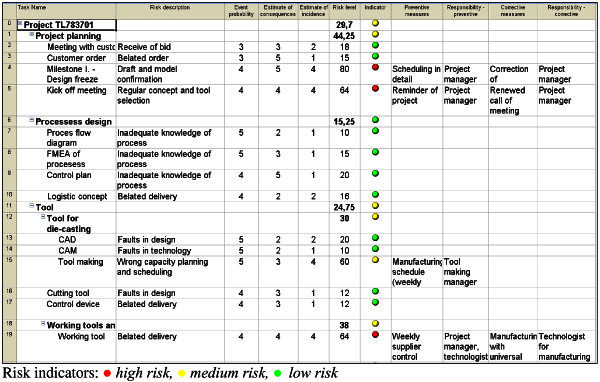
**Risk analysis table of project TL783701 activities in MS Project.**

The project manager copied the data from Table 8 to the template and the result is shown in Figure 8.

The advantage of the proposed template for project activities risk management is that the same software that is used for scheduling, resource and cost planning is also used for project activities risk management.

The proposed template has its limitations. If there are several possible risks within a particular activity, only the risk with the highest risk level is entered in the table in Figure 8.

The project manager, project team members and operators of activities can obtain the following data from Table 8:

- short definition of risks
- estimate of event probability
- estimate of event consequences
- estimate of event incidence
- risk level and risk indicator (in colour)
- responsibility for risk management
- link to a document with risks and measures described in detail.

The risk indicator colour visually warns the project manager and team members about the risk levels of individual activities and the expected preventive and corrective measures.

It can be seen from Figure 8 that this project has 4 high-risk activities, 2 medium-risk activities and 14 low-risk activities.

High-risk activities are connected to the costumer (design freeze) and to most important decision in the process of tool design – concept of the die-casting tool. Both activities have important role for other activities that follow them in the project of tool production and also for process of serial production of parts in the foundry.

For a comparison of individual project risk with other projects, the risk level of the whole project is useful. On the basis of (Paul 2002), we decided that the risk level of groups of activities and of the whole project would be calculated as an average risk level of all activities (the lowest level in project WBS). Of course, the average project-risk-level can only be statistical data, so it can be misleading if used uncritically. A project may have low average risk level, but it may contain high-risk-level activities. If a risk event occurs in these activities, it can be a serious threat to the completion of the project within the expected scope, time and costs.

In addition to the risk indicator, other indicators can be added to Table 8 to give warnings on other dangers related to project risks, e.g. delays, use of time-reserves, or exceeded actual costs in comparison with the planned ones.

## Conclusion

The article presents the problem of risk management in market-oriented projects (i.e., in product and service projects). We found that in cyclically recurring projects, the causes of activity risks are often similar and recurring.

A model for project activities risk management was developed. In our model, we added a third parameter to the well-known two-dimensional risk analysis method: problem incidence. This data can be estimated by evaluating already completed projects. Introduction of this additional parameter proved to be necessary in practical use, since it was required by both customers of project products and by project management system auditors.

If the estimated problem incidence is high and it does not get lower in future similar projects, it is obvious that the company is not effectively managing (eliminating) recurring problems. This is important data for the company management, which should urgently take appropriate measures. The goal of the proposed method includes gradually reducing the estimated problem incidences (target value is 1), and a gradual transition to two-dimensional risk analysis.

MS Project is often used for project management support in companies, so the employees of the Faculty of Mechanical Engineering in Ljubljana, Slovenia, together with our partner companies, made a new table (as an addition to the standard templates), which allows risk analysis with MS Project software. This template proved very useful in practice, because project managers could thus use the same software for planning and risk-management actions.

## Ethical approval

For this article no experimental research was carried out on humans or animals.

## References

[CR1] Cappels MT (2004). Financially focused project management.

[CR2] Dobie C (2007). A handbook of project management.

[CR3] Fleischer M, Liker KJ (1997). Concurrent engineering effectiveness: integrating product development across organisations.

[CR4] Goodpasture JC (2004). Quantitative methods in project management.

[CR5] Kendall IG, Rollins CS (2003). Advanced project portfolio management and the PMO.

[CR6] Kušar J, Rihar L, Duhovnik J, Starbek M (2008). Project management of product development. J mech eng.

[CR7] Paul RS (2002). Project risk management – a proactive approach.

[CR8] (2004). A guide to the project management body of knowledge.

[CR9] Risk management guide for DOD acquisition sixth edition. USA: Department of defense, USA; 2006. , accessed 17.5.2009

[CR10] Smith GP, Merritt MG (2002). Proactive risk management.

[CR11] Vargas VR (2008). Practical guide to project planning, auerbach publications.

